# To eat or to breathe? The answer is both! Nutritional management during noninvasive ventilation

**DOI:** 10.1186/s13054-018-1947-7

**Published:** 2018-02-06

**Authors:** Pierre Singer, Sornwichate Rattanachaiwong

**Affiliations:** 1 Department of General Intensive Care and Institute for Nutrition Research, Rabin Medical Center, Petah Tikva and Sackler School of Medicine, Tel Aviv University, Jabotinski St, Petah Tiqwa, 49100 Israel; 2 Division of Clinical Nutrition, Department of Medicine, Faculty of Medicine, Khon Kaen University, Khon Kaen, Thailand

**Keywords:** Enteral nutrition, Non-invasive ventilation, Aspiration

## Abstract

Treating respiratory distress is a priority when managing critically ill patients. Non-invasive ventilation (NIV) is increasingly used as a tool to prevent endotracheal intubation. Providing oral or enteral nutritional support during NIV may be perceived as unsafe because of the possible risk of aspiration so that these patients are frequently denied adequate caloric and protein intake. Newly available therapies, such as high-flow nasal oxygen (HFNO) may allow for more appropriate oral feeding.

Noninvasive ventilation (NIV) in the management of patients with respiratory failure reduces the work of breathing and may prevent further deterioration of the respiratory status while providing more comfort and less need for sedation than conventional mechanical ventilation via an endotracheal tube. It appears to be beneficial in both the acute and non-acute settings [1]. Regarding nutritional support in patients receiving NIV, a large observational French study showed that nearly 60% of patients were starved during the first 2 days of treatment and only 2.6% received enteral nutrition [2]. The Nutrition Day ICU audit of almost 10,000 patients worldwide, including 47% undergoing mechanical ventilation and 6.2% with NIV, found similar findings in that 40% of patients were starved during the first day of ventilation and 20% on the second day [3].

In addition to the fact that a large number of ICU patients do not receive nutritional support regardless of their admission etiology, the apparent reluctance to provide nutritional support during NIV may have several explanations. NIV support may not always be successful in preventing the requirement for endotracheal intubation and predicting which patients will deteriorate may not be straightforward. Thus, a nil-per-os order is commonly given in the event that intubation will be subsequently required. The presence of a nasogastric tube (NGT) may result in air leakage and compromise the effectiveness of NIV. While this problem may be circumvented with special NIV masks with a port for NGT, these are not always available and are costly. Positive pressure ventilation through a face mask also results in the stomach being dilated with air. The consequent gastric distention may adversely affect diaphragmatic function, further compromising the respiratory condition and resulting in endotracheal intubation. Patients who are allowed to have an oral diet may deteriorate when they remove the NIV in order to eat, thus resulting in deranged respiratory function. A retrospective observational study showed that receiving enteral nutrition during NIV was associated with a significantly higher rate of airway complications (53 vs 32%, *P* = 0.03) and longer NIV duration (16 vs 8 days, *P* = 0.02) compared to patients who did not receive enteral nutrition [4]. Failure of NIV to improve the patient is associated with increased mortality [5], explaining why physicians are reluctant to decrease the likelihood of success, for example, by prescribing enteral nutritional support. NIV is also used to prevent reintubation after extubation. During this period, oral intake is known to be as low as around 650 kcal/day [6]. After extubation, swallowing disorders (SD) may impair the return to normal food intake and moderate/severe SD are associated with a higher rate of regurgitation, pneumonia, length of stay, and mortality [7]. These reasons may result in the physician refraining from ordering oral/enteral feeding during the intermediate period preceding recovery. Interestingly, using high-flow nasal oxygen (HFNO) administration allowed complete oral alimentation in all the patients included in a recent study [8].

How hard should we try to achieve early and effective enteral nutrition (EEN)? According to the recent ESICM recommendations on early enteral nutrition, there is a clear advantage to EEN in decreasing infection complications in comparison to delayed enteral nutrition and to early parenteral nutrition [9]. The more a malnourished patient develops a calorie deficit, the worse the outcome [10] and malnourished patients should therefore be fed without delay to prevent an aggravation of their general condition. When considering the risk to benefit aspect, pre-existing malnourished patients benefit from nutritional therapy started within 24–48 h; they should receive sufficient protein and calories as soon as possible while the delivery of nutrition may be delayed in those well-nourished at baseline [11, 12].

Technically, NIV impairs oral and enteral feeding. If the patient is malnourished, HFNO should be considered to allow for the provision of calorie and protein requirements, or efforts should be made to feed the patient enterally. If the patient is well nourished, NIV can be initially prescribed without feeding, with reconsideration after a couple of days when an alternative therapy might be proposed. This alternative could be performed using an adapted NGT through a helmet limiting leaks. The use of parenteral nutrition has been suggested in patients with prolonged SD and in whom the reintroduction of a NGT may decrease the rate of success of swallowing rehabilitation. Although parenteral nutrition was thought to be associated with worse outcomes, recent studies demonstrate that it is excessive calories and not the route that are responsible for these complications [13]. To avoid over-nutrition, Siirala et al. [14] succeeded in measuring resting energy expenditure (REE) in patients with NIV with a canopy allowing the determination of a calorie target.

The large French observational study reported here stresses the fact that many patients with NIV are not fed in the ICU and that enteral feeding is associated with increased 28-day mortality, increased invasive ventilation needs, and more prolonged ventilation days compared to no nutrition [2]. These findings should not be the basis for letting our patients starve. Work of breathing accounts for a large part of the total energy expenditure (up to 25% in respiratory distress), and negative energy balance and exhaustion may be the reason for respiratory deterioration.

In conclusion, the dilemma should not be whether to breathe or to eat; instead, we need to use effectively the combination of new respiratory support devices with the appropriate route for nutritional therapy. Prospective studies taking into account the nutritional condition of the patient and the ability to be treated with HFNO instead of NIV should be planned.
